# Lessons learned: from mentored to mentor

**DOI:** 10.1172/JCI167444

**Published:** 2023-01-17

**Authors:** Joseph Heitman

**Affiliations:** Department of Molecular Genetics and Microbiology, Duke University School of Medicine, Durham, North Carolina, USA.

## Abstract

This Viewpoint was written in association with the 25th anniversary of the American Society for Clinical Investigation’s (ASCI’s) Stanley J. Korsmeyer Award, which honors the highest standards of scientific excellence, meritorious research, intellectual integrity, and the mentoring of future life-science researchers. In 2018, the award recognized Joseph Heitman (Figure 1), for his key contributions to our understanding of how eukaryotic microbial pathogens evolve, cause disease, and develop drug resistance and his discovery of TOR and FKBP12 as targets of the immunosuppressive chemotherapeutic drug rapamycin. Dr. Heitman has mentored numerous undergraduates, medical students, graduate students, and postdoctoral and medical fellows, many of whom have developed independent careers in medicine and basic biomedical research.

*I have been the beneficiary of a series of accidents, as are we all.*

(With apologies and homage to Kurt Vonnegut.) 

It is often said that one must first learn before they can teach, and in the realm of mentoring, the mentor often learns as much as those who are mentored. I started as an assistant professor on September 1, 1992, and now 30 years hence reflect on experiences and lessons learned. At the outset, I knew the thrill of discovery-driven science, but I had not anticipated how fulfilling mentoring would prove to be.

I learned from exceptional role models in science, from undergraduate research experiences with Gus Fried, Kan Agarwal, and Malcolm Casadaban at the University of Chicago to graduate work in biology with Peter Model and Norton Zinder at The Rockefeller University as an MD-PhD student. Thereafter, a leave of absence from medical school provided a formative postdoctoral experience with Mike Hall at the Biozentrum and Rao Movva at Sandoz in Basel, Switzerland. This was followed by a return to New York City to complete medical school in June 1992 and then a move to Duke University as an assistant professor in September 1992.

My laboratory was founded on a research program exploring molecular targets and mechanisms of action of natural products with antimicrobial activity. The focus was on immunosuppressive natural products: cyclosporin A, FK506/tacrolimus, and rapamycin/sirolimus (1). The approach harnessed the power of yeast genetics and capitalized on the intrinsic antifungal activity of these natural products made by soil microbes to inhibit competing microbes. My first graduate student, Michael Lorenz, initially focused on defining genetically the target of FKBP12-rapamycin, the TOR1 and TOR2 kinases. Mike’s studies contributed to the defining of the FKBP12-rapamycin interaction domain on TOR and provided evidence that mutations conferring rapamycin resistance disrupted binding (2). He went on to adduce evidence that the TOR pathway responds to nutrients and controls cellular responses. Around this time, Gerry Fink’s lab at the Whitehead Institute had discovered that nutrient limitation stimulated yeast cells to undergo a dimorphic transition from yeast to pseudohyphae. Fascinated by this, Mike wrote to Marcelle Grenson in Belgium to request the yeast strain, Σ1278b. I remember vividly the afternoon we spent looking through the microscope, captivated by the model budding yeast that produced elaborate ruffled colonies decorated with luxuriant pseudohyphae. We were hooked! We launched a new project to focus on how cells sense nutrients. Mike discovered a novel role for the ammonium permease Mep2 as a transceptor for the pathway (3). We now know that the mammalian Rh antigens are physical and functional homologs of the yeast ammonium permeases. Mike further discovered that the Gα protein Gpa2 plays a central role in a second nutrient-sensing pathway activated by the GPCR Gpr1, which senses fermentable sugars and activates cAMP signaling to control cell physiology (4). Thus, from the first student in the lab sprung a well of projects and ideas. Mike went on to study how *Candida albicans* responds transcriptionally following phagocytosis by macrophages with Gerry Fink and is now a full professor at the UTHealth Houston McGovern Medical School (University of Texas, Houston).

In late 1993, John Perfect, now Chief of the Division of Infectious Diseases (ID) at Duke, called my office to discuss a possible collaboration on the pathogenic yeast *Cryptococcus*. John sent two people to work in my lab, ensuring this would be a productive new direction. The first was Audrey Odom John, then a Duke undergraduate who had worked in John’s lab. Audrey spent 3 years working in my lab and successfully deleted the gene encoding the catalytic subunit of the phosphatase calcineurin, the target of the immunosuppressive and antifungal action of cyclosporin and FK506 (5). Audrey matriculated in the Duke MD-PhD program and then completed her clinical training in pediatric ID at the University of Washington. She is currently the Pediatric ID Division Chief at Children’s Hospital of Philadelphia.

The second person John sent to my lab was J. Andrew (Andy) Alspaugh, MD, an ID fellow training at Duke. Andy wanted to work with John, but John convinced Andy to take a risk and work with a new assistant professor just getting started with ID research. Andy had worked on the antifungal activity of nitric oxide in *Cryptococcus* with Don Granger during the third year of medical school at Duke and had trained with Pat Pukkila at the University of North Carolina at Chapel Hill as an undergraduate. Thus, Andy arrived with a great deal of prior research experience. Bill Courchesne and colleagues had recently published a paper about a novel Gα protein in *Cryptococcus* and suggested it might be involved in pheromone sensing. But, based on Mike Lorenz’s recent results studying the orthologous Gα protein in *Saccharomyces*, we surmised it might be instead involved in nutrient-sensing cAMP signaling. Andy successfully disrupted the *GPA1* gene by biolistic transformation and homologous recombination and, through elegant phenotypic analysis, demonstrated that it was required for cAMP signaling and activation of synthesis of two virulence factors: capsule and melanin. As a consequence, the *gpa1* mutant strains were severely attenuated in virulence in animal models (6). Andy subsequently joined the faculty at Duke, where he has become a full professor, vice chair of academic affairs in the Department of Medicine, and director emeritus of the Marine Biological Lab (MBL) Molecular Mycology Course in Woods Hole.

I first met Tim James in 1998 when he was a graduate student working with Rytas Vilgalys at the Duke Department of Biology. Rytas had suggested that Tim invite me to serve on his PhD thesis committee, and over the next several years I am certain that I learned much more from Tim than he did from me. Tim’s focus was on the structure and evolution of mating-type loci in basidiomycete fungi, and he was rapidly cloning these regions via their linkage to a flanking gene encoding a mitochondrial protease. What Tim and Rytas imparted was a deep love and appreciation for an evolutionary perspective and considering fungi in a phylogenetic context. Moreover, Tim was interested in transitions between outcrossing and inbreeding/selfing in fungi and how changes in mating-type locus configuration contribute to transitions in modes of sexual reproduction. On a flight back from one of the *Cryptococcus* genome sequencing meetings in 1999, Rytas sketched out on the back of an envelope the species most closely related to the *Cryptococcus* pathogenic species complex. These discussions formed the foundation for our longer-term research focused on the structure, function, and evolution of mating-type loci. These studies were conducted by postdoctoral fellows Klaus Lengeler and James Fraser, who defined the complete sequence of the *Cryptococcus* mating-type locus and its evolutionary trajectory (7, 8), and by fellow Christina Hull and graduate student Rob Davidson, who discovered the fungal mating type determinants (Sxi1α and Sxi2a) and genetically defined their roles in the sexual cycle (9). As a fellow in the lab, Xiaorong Lin discovered unisexual reproduction as a novel mode of selfing in human fungal pathogens that contributes to clonal population structures and production of infectious propagules (10). Tim is now the Lewis E. Wehmeyer and Elaine Prince Wehmeyer Professor in the Taxonomy of Fungi at the University of Michigan and the president of the Mycological Society of America; Klaus is a scientist at the Carlsberg labs in Denmark; James is a professor at the University of Queensland; Christina is a professor at the University of Wisconsin–Madison; Rob is a senior scientist at Johnson & Johnson; and Xiaorong is the Gene E. Michaels Distinguished Professor in Medical Mycology at the University of Georgia.

As a highly successful fellow in the lab, Alex Idnurm worked on a panoply of projects from fungal light sensing to basal fungal sex loci. During this time, he served as the mentor for an exceptionally talented undergraduate, Felicia Walton Pagliuca. Together, they published four papers on fungal morphogenesis, insertional mutagenesis, and virulence factor production. Felicia received a Marshall Scholarship and moved to the University of Cambridge where she worked with Jonathon Pines, who was her advisor. From there she moved to Harvard as a fellow with Doug Melton. Felicia discovered how to differentiate human stem cells into functional β cells and published these landmark studies in *Cell* (11). She started at Harvard Business School, thinking she might want to start a company someday. But during a leave of absence, Felicia and Doug cofounded Semma Therapeutics. Some years later Semma Therapeutics was sold to Vertex for $950 million dollars; Semma Therapeutics is maintained within the larger company as a subsidiary, which Felicia and Doug continue to manage. Just over a year ago, the first patients with type 1 diabetes were treated with the novel cell-based therapy that Felicia helped to develop, and the initial results are exceptionally promising. Alex is now a professor at the University of Melbourne and continues to excel with his research on fungal genetics and pathogenesis.

Our research took an unexpected direction during Cecelia Shertz Wall’s time in the lab as a graduate student. Cecelia’s project was to isolate FK506-resistant mutants of the basal human fungal pathogen *Mucor*, based on the finding by Soo Chan Lee, a fellow in the lab, that inhibition of calcineurin by FK506 blocked the dimorphic transition from yeast to hyphae required for infection. Cecelia and Soo Chan discovered two classes of FK506-resistant isolates, one involving Mendelian mutations in the known drug targets FKBP12 and calcineurin A and B, and a second that conferred transient drug resistance that reverted during passage without the drug. Cecelia discovered that this second class of antimicrobial drug–resistant isolate results from RNAi silencing of the gene encoding the FKBP12 drug target via epimutation. Silvia Calo joined the lab as a fellow and defined the RNAi pathways and components that are responsible for epimutation, generalized this to another fungal species, and explored the effect in a whole-genome context (12). Epimutations have been discovered in plants with flower pattern mutations and in humans with defects in mismatch repair who have Lynch syndrome. We now appreciate that epimutations can be conferred by RNAi, DNA methylation, or heterochromatin, all of which lead to defects in gene expression that result in drug resistance, aberrant development, or disease.

For the past 25 years I have taken an annual sojourn to teach the molecular mycology course at the MBL in Woods Hole. I was invited to visit in 1998 by one of the original founders of the course, Aaron Mitchell. I was then invited to return each year as an instructor in residence. More than perhaps any other activity, this course has forged the field of medical mycology into the vibrant and interactive community that it is today. From my own perspective, the opportunity to learn by teaching the next generation of leaders in the field has been inspirational. On another level, this annual sojourn serves as a sabbatical, taken one or two weeks at a time.

My service as chair of the Department of Molecular Genetics and Microbiology at Duke University commenced on September 1, 2009. That same day, Sheng Sun arrived to start as a new post-doctoral fellow in the lab. Fortuitously, Sheng had been training in the same lab as Tim James at McMaster University with JP Xu. Tim urged Sheng to consider my lab for further training, and Tim also urged me to accept Sheng. Hiring Sheng Sun is one of the best lab management decisions I have ever made. Sheng began as a postdoctoral fellow and was then promoted to senior research associate and then to research-track professor. Among the many discoveries that he has contributed over the past 13+ years, his recent discovery of mutations in a novel GTP exchange factor that enhance and enable fungal unisexual reproduction, including of lineages and species with restricted fertility, is of particular significance (13–15). If there is a secret to having been able to run a successful research lab while also having an administrative role, it has been to have outstanding senior support in the lab. This has included Sheng, my long-term lab manager Anna Averette, and my administrative coordinator Melissa Palmer.

In closing, to summarize mentoring lessons learned over the past 30 years: delegate responsibility, avoid micromanagement, inspire life-long learning, and commit to support and mentor trainees, not only for the time they are in your lab, but for their entire careers.

We strive to do the best science we can and, in so doing, to train the best scientists that we can train.

## Figures and Tables

**Figure 1:**
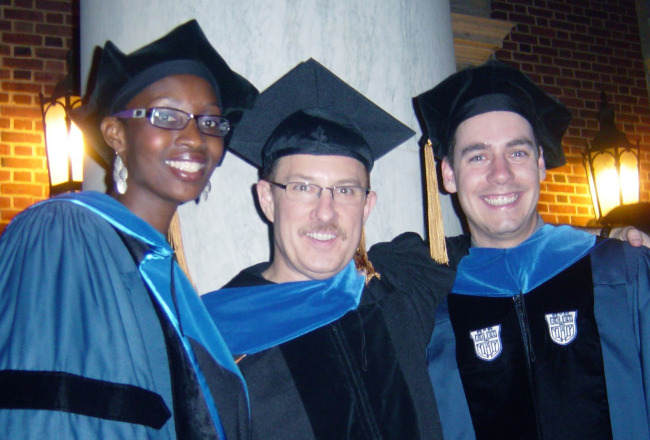
Joseph Heitman, at a Duke University graduation ceremony with two graduating PhD students, Keisha Findley and Edmond Byrnes.

## Version 1 01/17/2023

Electronic publication
